# A Rapid, Hippocampus-Dependent, Item-Memory Signal that Initiates Context Memory in Humans

**DOI:** 10.1016/j.cub.2012.10.055

**Published:** 2012-12-18

**Authors:** Aidan J. Horner, David G. Gadian, Lluis Fuentemilla, Sebastian Jentschke, Faraneh Vargha-Khadem, Emrah Duzel

**Affiliations:** 1 Institute of Cognitive Neuroscience, University College London, 17 Queen Square, London WC1N 3AR, UK; 2 Institute of Neurology, University College London, Queen Square, London WC1N 3BG, UK; 3 Institute of Child Health, University College London, 30 Guilford Street, London WC1N 1EH, UK; 4 Department of Paediatric Neuropsychology, Great Ormond Street Hospital for Children NHS Foundation Trust, Great Ormond Street, London WC1N 3JH, UK; 5 Cognition and Brain Plasticity Unit, Institute of Biomedicine Research of Bellvitge, 08908 Barcelona, Spain; 6 Basic Psychology Department, University of Barcelona, 08035 Barcelona, Spain; 7 Institute of Cognitive Neurology and Dementia Research, Otto von Guericke University, 39120 Magdeburg, Germany; 8 German Center for Neurodegenerative Diseases (DZNE), Magdeburg, 39120 Magdeburg, Germany

## Abstract

The hippocampus, a structure located in the temporal lobes of the brain, is critical for the ability to recollect contextual details of past episodes. It is still debated whether the hippocampus also enables recognition memory for previously encountered context-free items. Brain imaging [1, 2] and neuropsychological patient studies [3, 4] have both individually provided conflicting answers to this question. We overcame the individual limitations of imaging and behavioral patient studies by combining them and observed a novel relationship between item memory and the hippocampus. We show that interindividual variability of hippocampal volumes in a large patient population with graded levels of hippocampal volume loss and controls correlates with context, but not item-memory performance. Nevertheless, concurrent measures of brain activity using magnetoencephalography reveal an early (350 ms) but sustained hippocampus-dependent signal that evolves from an item signal into a context memory signal. This is temporally distinct from an item-memory signal that is not hippocampus dependent. Thus, we provide evidence for a hippocampus-dependent item-memory process that initiates context retrieval without making a substantial contribution to item recognition performance. Our results reconcile contradictory evidence concerning hippocampal involvement in item memory and show that hippocampus-dependent mnemonic processes are more rapid than previously believed.

## Highlights

► Whether the hippocampus is needed for item as well as context memory is unclear ► We tested behavior and function in a large patient group with hippocampal atrophy ► We identified a new rapid hippocampus-dependent functional signal to familiar items ► This signal is not needed for item memory but initiates context recollection

## Results

An important goal in memory research is to map the functional organization of a cognitive process onto anatomy. This has proven particularly controversial for the hippocampus, a structure located within the medial temporal lobes (MTL), and its relationship to the functional components of recognition memory [5, 6]. Dual-process models of recognition memory distinguish between two components, namely, familiarity with items and recollection of context [7–9]. To date, it is still unresolved whether the hippocampus is selectively required for context memory or also for item memory.

Behavioral studies of patients with selective hippocampal lesions have produced conflicting results. Whereas some studies have shown selective deficits in context memory following hippocampal injury [3, 9, 10], others have also shown item-memory deficits [4, 11–13]. Lesion-behavior studies, however, suffer from a difficulty in unambiguously dissociating between memory processes (e.g., item versus context memory), making it difficult to conclude that a given process is functionally preserved.

Another influential line of research using functional imaging has also provided conflicting results. Some studies have shown selective context-related signals in the hippocampus [1, 14]; others have shown both item and context signals [2, 15]. Imaging studies, however, do not allow conclusions about causality: the presence of an item signal in the hippocampus does not allow the conclusion that the hippocampus is critical for item-memory performance (see [16] for a review of patient and imaging data).

By combining a lesion-behavior study with functional imaging, we overcame the limitation of each method alone and sought to identify a causal relationship between hippocampal integrity and functional as well as behavioral indices of item and context memory. We used the largest sample of patients with selective hippocampal lesions to date to participate in a functional imaging experiment. Patients had varying degrees of selective hippocampal injury following an early-life hypoxic-ischemic episode.

**Figure 1:**
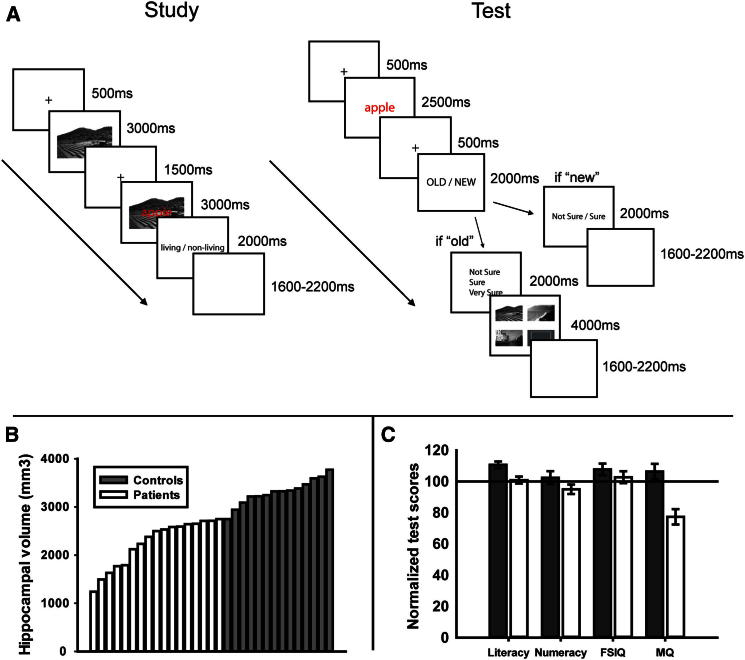
Experimental Design, Hippocampal Volumes, and Neuropsychological Test Scores for Patients and Controls (A) Study and test phase trial sequences for the item and context memory task. At study, word-scene pairs were presented, and participants were required to judge whether the word denoted a living or nonliving object (see Supplemental Information). At test, old and new words were presented, and participants were required to judge the old/new status of the word. If they responded “new,” they rated their confidence with the options “not sure” or “sure” (see Supplemental Information for analyses of confidence judgments). If they responded “old,” they rated their confidence for the upcoming context decision with the options “not sure,” “sure,” or “very sure” and then chose the scene originally paired with the word from three alternatives (plus a blank square if they believed the scene was not presented). (B) Hippocampal volumes across the participant group showing controls (dark gray) and patients (white). (C) Literacy, numeracy, full-scale IQ (FSIQ), and memory quotient (MQ) neuropsychological test scores. Patients showed comparable performance to controls and to the standard population mean (i.e., 100 ± 15) in terms of literacy, numeracy, and FSIQ but showed a clear deficit in MQ. Error bars show ±1 SEM for each condition.

We recorded magnetoencephalography (MEG) while 17 patients and 14 controls participated in an item (words) and context (scenes) associative recognition-memory paradigm (Figure 1A). During a study (or encoding) phase, words were superimposed over visual scenes. Memory for the item and its paired context were later tested using an old/new recognition judgment (item memory) and a scene three-alternative forced choice (context memory), respectively. Due to the graded severity of pathology in this large sample, we were able to utilize an extensive variability of hippocampal integrity across all patients, as well as controls (Figure 1B). We therefore adopted a novel correlative approach to assessing hippocampus dependency (see Supplemental Information available online). Behaviorally, we correlated hippocampal volume with measures of both item and context memory. For the imaging data, we adopted an approach that was blind to differences between context hits versus misses by correlating hippocampal volume initially with overall item recognition signals (i.e., collapsed across context hits and misses). In a second step, we assessed whether these memory signals were selectively related to item or context memory or incorporated both components.

**Figure 2:**
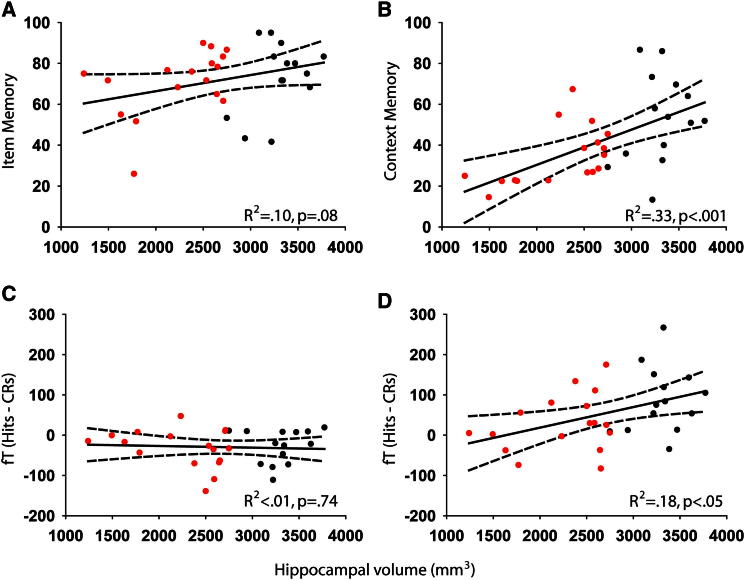
Correlating Hippocampal Volume with Memory Performance and Magnetoencephalographic Effects Correlation analyses between bilateral hippocampal volume and item memory (Pr) (A), context memory (conditional context hits) (B), the 300–350 ms occipitotemporal effect (hits – CRs) (C), and the 350–400 ms frontotemporal effect (hits – CRs) (D) across all participants. Solid lines represent the line of best fit, and dashed lines represent 95% confidence intervals. Patients are highlighted in red, controls in black.

Participants’ item memory, judging whether a word at test was previously presented at study, was high with patients and controls showing >83% hits (old items classified as old) and <15% false alarms (FAs: new items classified as old; see Table S1). Using a corrected hit rate (Pr = hits − FAs) measure of discriminability, we saw only a marginal correlation with hippocampal volume: *R*^2^ = 0.10, df = 29, p = 0.08 (see Figure 2A and Supplemental Information for correlations within the patient group only). Furthermore, no significant item-memory difference between patients and controls was seen: *t*(29) = 1.25, p = 0.22. Thus, we could not find firm evidence for a relationship between hippocampal volume and item-memory performance.

Context memory performance was calculated by dividing the number of context hits (correct scene selection) by the number of item hits for each participant. Context memory significantly correlated with hippocampal volume: *R*^2^ = 0.33, df = 29, p < 0.001 (Figure 2B), and was impaired in patients (33.1% context accuracy) relative to controls: 54.9%, *t*(29) = 3.57, p < 0.01. Finally, patients did not perform significantly above chance: *t*(16) = 0.04, p = 0.97 [controls relative to chance: *t*(13) = 3.99, p < 0.01]. Thus, we found strong evidence to suggest hippocampal volume was related to context memory performance. Behaviorally, we therefore saw a clear dissociation, with the hippocampus selectively contributing to context, but not item-memory performance (see Supplemental Information for a direct comparison of the relationship between hippocampal volumes and item and context memory, as well as analyses of subjective confidence ratings).

Next, we assessed whether our findings were selective to the hippocampus or also extended to extrahippocampal regions of the MTL that are known to be involved in recognition memory (for a review, see [5]). Hippocampal volumes were not correlated with the volume of the parahippocampal region, indicating that hippocampal injury was anatomically specific. Furthermore, unlike hippocampal volumes, the volume of the parahippocampal region correlated significantly with item but not context memory (see Supplemental Information).

**Figure 3:**
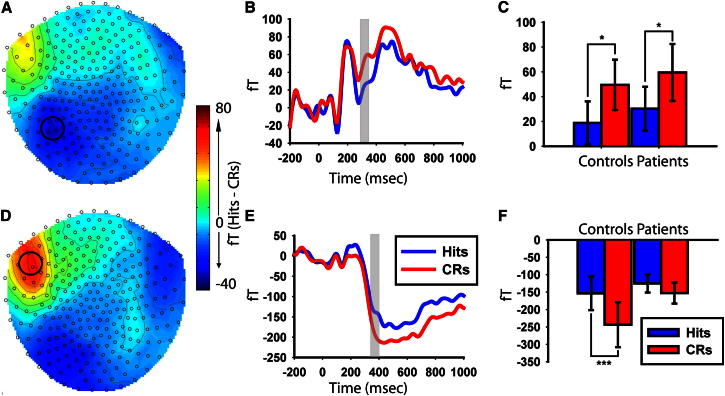
Topographies, Event-Related Fields, and Time Windows of Interest for the Magnetoencephalographic Effects Topographies (A and D), event-related fields (ERFs) collapsed across patients and controls (B and E), and time-window analyses (C and F) for the clusters revealed in the 3D SPM analysis (see Figure S1). The occipitotemporal 300–350 ms effect (A–C) is shown; the frontotemporal 350–400 ms effect (see Supplemental Information for analysis of later 700–750 ms effect) (D–F) is shown. Topographies represent the difference in fT between hits and CRs (collapsed across controls and patients) at the mid time point in the 50 ms time window, with the black circles highlighting the sensors selected for further analyses. The ERF plots show the ERFs for hits and CRs averaged across the peak sensors highlighted in the topographies, with time windows for further analyses highlighted in gray. The time-window analyses represent the average (fT) within the 50 ms highlighted in gray in the ERF plots, plotted separately for controls and patients. Error bars show ±1 SEM for each condition; ^∗∗∗^p < 0.001, ^∗∗^p < 0.01, ^∗^p < 0.05.

**Figure 4:**
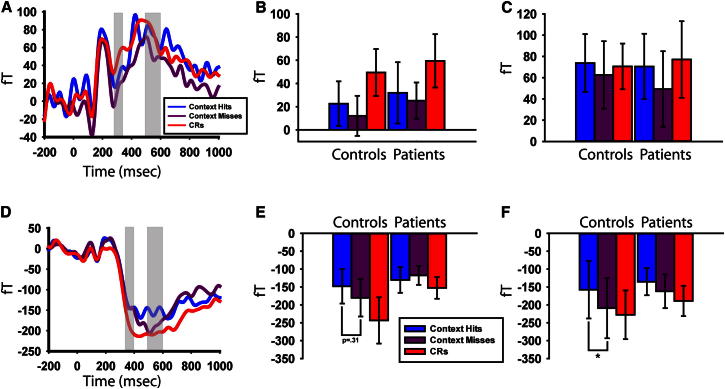
Event-Related Fields and Time Windows of Interest across Context Hits, Context Misses, and Item CRs for the Two Magnetoencephalographic Effects (A and D) ERFs collapsed across patients and controls and time-window analyses for (B and E) the early time windows (300–350 ms occipitotemporal effect and 350–400 frontotemporal effects) and (C and F) the 500–600 ms time window. The occipitotemporal effect (A–C) is shown; the frontotemporal effect (D–F) is shown. ERF plots show ERFs for context hits, context misses, and item CRs (collapsed across controls and patients), with time windows for further analyses highlighted in gray. The time-window analyses represent the average (fT) within the 50 ms or 100 ms highlighted in gray in the ERF plots, plotted separately for controls and patients. Error bars show ±1 SEM for each condition; ^∗^p < 0.05.

In our MEG data, we looked across all sensors and time points (see Supplemental Information for analysis details) for significant differences in event-related fields (ERFs) between item-memory hits and correct rejections (CRs: new items identified as new). Two distinct effects were seen. The first was over left occipitotemporal sensors from 300 to 350 ms (Figure S1A). This presented as a large deviation for both hits and CRs relative to baseline from 150 ms onward, with a diminished effect for hits relative to CRs (Figure 3). This occipitotemporal effect failed to show a relationship with hippocampal volume: *R*^2^ < 0.01, df = 29, p = 0.74 (Figure 2C). Furthermore, this effect was present in both the patients: *t*(16) = 2.51, p < 0.05, and controls: *t*(13) = 2.83, p < .05 [hits versus CRs difference for patients versus controls: *t*(29) = 0.09, p = 0.93]. Finally, when splitting control participants’ item hits into context hits and context misses, no context differences were seen over occipitotemporal sensors, either in the 300–350 ms time window (*t*’s < 0.77, p’s > 0.46) or in a later 500–600 ms time window (*t*’s < 0.86, p’s > 0.40), when context effects typically emerge in EEG (Figure 4) [17]. As such, the early transient occipitotemporal effect is likely a neural signature of the behavioral item-memory effect.

The second MEG effect emerged over left frontotemporal sensors from 350 to 400 ms (Figure S1B) and persisted for 400 ms. This frontotemporal effect presented as a large deviation for both hits and CRs relative to baseline (in the opposite direction to the occipitotemporal effect) with diminished amplitude for hits relative to CRs (Figure 3) and correlated with hippocampal volume: *R*^2^ = 0.18, df = 29, p < 0.05 (Figure 2D). Furthermore, whereas the effect was seen in the controls: *t*(13) = 4.24, p < 0.001, it was absent in the patients: *t*(16) = 1.63, p = 0.12 [hits versus CRs difference for patients versus controls: *t*(29) = 2.31, p < 0.05]. Thus, the frontotemporal effect closely mirrored the behavioral context memory results (see Supplemental Information for a direct comparison of hippocampal correlations with the frontotemporal and occipitotemporal effects).

Further analyses of the frontotemporal effect revealed a novel functional relationship between item and context memory. First, we found no amplitude difference between context hits and context misses between 350 and 400 ms for the control participants: *t*(13) = 1.05, p = 0.31 [patients: *t*(16) = 0.45, p = 0.66; Figure 4], indicating that this time window reflected an item-memory effect. Instead, controls showed a context hits versus context misses amplitude difference in a later time window of the frontotemporal effect, between 500 and 600 ms: *t*(13) = 2.55, p < 0.05, a period during which context effects typically emerge in EEG studies [17, 18]. This effect was absent in patients: *t*(16) = 0.82, p = 0.41, confirming that, like the early frontotemporal item effect, it is also hippocampus dependent (the size of the context effect, however, did not correlate with hippocampal volume: *R*^2^ = 0.07, df = 29, p = 0.15). No further time period over occipitotemporal or frontotemporal sensors showed a significant context hits versus context misses effect, suggesting context modulation was specific to the 500–600 ms time window over frontotemporal sensors (see Supplemental Information).

Thus, our data show the existence of two topographically contiguous hippocampus-dependent processes: an early (350–400 ms) item-memory process followed by a later (500–600 ms) context-memory process. Importantly, this hippocampus-dependent item-memory process was temporally distinct from the aforementioned occipitotemporal item-memory effect. Thus, the frontotemporal item-memory effect reflects a previously unreported item-memory process that is already hippocampus dependent and evolves into a context-memory process. Further within-subject analyses of trials with large and small item-memory effects confirm a temporal dependency between the 500–600 ms context-memory effect and the frontotemporal, but not the occipitotemporal, item-memory effect (see Supplemental Information).

## Discussion

Our data show that hippocampus-dependent memory processes are rapidly brought online at 350–400 ms, which is ∼150 ms earlier than previously suggested [17]. The existence of an early hippocampus-dependent effect presenting first as an item-memory effect and subsequently evolving into a context-memory effect could be seen as providing evidence for a hippocampal contribution to both item and context memory. However, the dissociation between our functional and behavioral measures questions this interpretation. Despite the fact that this same frontotemporal effect was absent in our patients, their item memory was not impaired. The early hippocampus-dependent frontotemporal item effect would therefore not appear to causally drive behavioral item-memory performance. Instead, item-memory performance appeared to depend on the volume of the parahippocampal region.

Our combined lesion-behavior and functional imaging methodologies help reconcile seemingly contradictory findings regarding the involvement of the hippocampus in item memory in previous studies [2, 15]. We have identified a functionally hippocampus-dependent item-memory process that behaviorally shows only a trend (Figure 2) toward a correlation with item-memory performance. These data indicate that hippocampus-dependent item-memory signals may not be critical for item-memory performance per se but may set the stage for successful context recollection. Hence, functional imaging studies that report hippocampal item-memory signals do not contradict the possibility that the hippocampus is critical for context memory, but not for item memory, as suggested by a number of previous studies [2, 15, 19]. At the same time, our results also show that a purely functional, i.e., imaging based, dissociation of item- and context-memory processes, as suggested by a number of previous studies [1, 14], may be an oversimplification.

What type of process does the early hippocampus-dependent item-memory signal denote? We suggest that this effect reflects the rapid recruitment of a memory process that requires the hippocampus and enables subsequent context retrieval. This proposal is consistent with recent evidence for an early intracranial hippocampal signal [20] (though we note the authors found context effects earlier than in the present study). Thus, although context information may be retrieved from 500 ms onward, hippocampus-dependent mnemonic processes that set the stage for subsequent context retrieval are initiated much earlier than previously suggested. One possibility is that our early hippocampus-dependent effect reflects the onset of pattern completion processes thought to be critical for the retrieval of a complete hippocampal memory engram (in this case, a word-scene association) following the presentation of a partial cue only (i.e., a word) [21, 22]. Thus, context effects would begin to emerge only later (i.e., 500–600 ms) following pattern completion, allowing for the phenomenological experience of recollection. This interpretation does not rule out the possibility that such an item-initiated pattern-completion process also provides some benefits for item memory, as suggested by the trend for a correlation between hippocampal volume and item-memory performance (Figure 2).

We propose item-memory performance is primarily supported by an early (300–350 ms) process outside of the hippocampus (e.g., perirhinal cortex). This item signal rapidly propagates to the hippocampus, initiating a pattern-completion process from 400 ms onward. Completion of such an item-initiated pattern-completion process at 500–600 ms results in the retrieval of context memory (and possibly further benefits item memory).

Our results therefore reconcile seemingly contradictory evidence concerning the role of the hippocampus in item memory and provide new insight into the timing of such mnemonic processes. These observations show that hippocampus-dependent processes involved in episodic memory, that is, the “reliving” of past events, begin much earlier than we previously thought and can initially be devoid of context information.

## Experimental Procedures

### Participants

Seventeen patients and fourteen controls participated (see Figure 1, Supplemental Information, and [23, 24] for patient and control details).

### Medial Temporal Lobe Volume Data

Whole-head 3D T1-weighted FLASH images (1 mm^3^) were acquired on a 1.5T Siemens Avanto system. Bilateral hippocampi were manually segmented using MEDx software and subsequently corrected for total intracranial volume. All volumes are reported in mm^3^ and relate to the mean across bilateral hippocampi (patient group: 2,264 mm^3^, SD 496; control: 3,295 mm^3^, SD 298). Extrahippocampal MTL volumes were estimated, using automated FreeSurfer analyses [25]. We used the parahippocampal region as defined in [26] because this covers a large portion of MTL volume, including the parahippocampal gyrus, as well as portions of the perirhinal cortex.

### Materials

For each participant, 120 concrete nouns (42% living, 58% nonliving), with a Kucera-Francis frequency of 2–17, were randomly assigned to one of two categories relating to the old and new conditions, respectively. Sixty grayscale images of scenes (500 × 300 pixels, 50% indoor, 50% outdoor) were randomly assigned to each old word, creating 60 word-scene pairs.

### Procedure

The experiment consisted of six study-test cycles. At study, participants were presented with word-scene pairs and required to perform a living/nonliving judgment on the word. At test, old/new words were presented, and participants performed an old/new item recognition judgment (item memory) and a further confidence judgment. If they responded “old” they also performed a three-alternative forced-choice source-memory test, choosing which scene was originally paired with the word (context memory) (see Figure 1A and Supplemental Information for details of trial sequence).

Participants were seated upright with their head underneath the helmet of an MEG machine. Stimuli were back projected onto a screen approximately 1 m in front of them, with images subtending approximately 6° of horizontal and vertical visual angle.

### MEG Acquisition and Analysis

MEG was recorded in a magnetically shielded room using a 275-channel CTF MEG system in third-order gradiometer configuration at a sampling rate of 600 Hz. Analyses were conducted using SPM8 (http://www.fil.ion.ucl.ac.uk/spm) (see Supplemental Information for MEG data preprocessing). To search for sensor-level ERF differences between conditions across all sensors and time points, we adopted a mass univariate approach, in which F tests were performed at every point in a 3D image of channel space and time (as detailed in Supplemental Information and [27]). We searched across all voxels for significant differences (p < 0.001 uncorrected) between hits and CRs regardless of patient status (i.e., collapsed across patients and controls). We subsequently focused our analyses on time windows and sensors of interest identified by this main effect to look for correlations between ERFs and hippocampal volume, as well as differences between controls and patients.

## Supplemental Information

Document S1. Supplemental Discussion, Figures S1–S3, Tables S1 and S2, and Supplemental Experimental Procedures
