# Supplementary material for: A Rapid, Hippocampus-Dependent, Item-Memory Signal that Initiates Context Memory in Humans
Source: Curr Biol. 2012 Dec 18;22(24):2369–74. doi: 10.1016/j.cub.2012.10.055 (PMC3661975; doi:10.1016/j.cub.2012.10.055)
Supplement: Document S1. Supplemental Discussion, Figures S1–S3, Tables S1 and S2, and Supplemental Experimental Procedures [file mmc1.pdf]

**Current Biology, Volume *22***
**Supplemental Information**

# A Rapid, Hippocampus-Dependent, Item-Memory Signal that Initiates Context Memory in Humans

**Aidan J. Horner, David G. Gadian, Lluis Fuentemilla, Sebastian Jentschke, Faraneh Vargha-Khadem, and Emrah Duzel**

## Supplemental Information Inventory

**Supplemental Discussion**

**Study Phase.** Analysis of study phase task performance
**Further Behavioural Analyses.** Higher-order interactions, patient-only correlations, parahippocampal volume analyses
**Confidence Judgments.** Analysis of confidence judgments
**3D SPM Analyses.** Analyses of 3D SPMs

**Supplemental Tables and Figures**

**Tables S1 and S2**
**Figures S1–S3**

**Supplemental Experimental Procedures**

**Participants.** Details of patient and control participants
**Procedure.** Further details of experimental procedure
**Medial Temporal Lobe Volume Data.** Details of methods for estimating MTL volumes
**MEG Data Preprocessing.** Epoching and filtering information for MEG data
**3D SPM Analysis.** Analyses across all timepoints and sensors
**Correlative Analysis Approach.** Rationale for hippocampal correlation approach

## Supplemental Discussion

### Study Phase

Accuracy for the living/non-living judgment during Study was high (mean 78%; SD 9%), with no relationship between hippocampal volume and performance, $R^2$=.07, d.f.=29, p=.15. Furthermore, no difference was seen between patients (mean 76%; SD 9%) and controls (mean 81%; SD 10%), $t(29)$ = 1.52, p=.14). As such, participants, regardless of hippocampal volume, were attentive to the word stimuli during the Study phase. Finally, no correlation was seen between reaction time in the living/non-living judgment (mean 818ms; SD 158ms) and hippocampal volume, $R^2$<.01, d.f.=29, p=.91, with no difference between patients (mean 804ms; SD 173ms) and controls (mean 831ms; SD 137ms), $t(29)$ = .49, p=.63.

### Further Behavioural Analyses

Table S1 presents performance on the item and context memory task. The analyses reported in the main manuscript support the claim that context memory is selectively hippocampus-dependent. As a follow-up we assessed whether the intact item memory and impaired context memory effects significantly differed from one another. Firstly, comparing the hippocampal correlation analyses, we converted the item and context memory R-values to Fisher Z-scores and found a significant difference between the two correlations, (Z=1.75, p<.05 one-tailed). Confirming this analysis, an 2x2 (Item vs. Context x Patients vs. Controls) ANOVA revealed a significant interaction, $F(1, 29)$ = 7.39, p<.05. Thus, the hippocampus showed a clear relationship with context but not item memory.

The correlational analyses across hippocampal volumes necessarily assumed a purely quantitative relationship between hippocampal volume and functional integrity across the patient and control groups. The patient vs. control comparisons do not suffer from this issue, however we also performed hippocampal correlations within the patient group alone. These analyses revealed a marginal positive correlation between hippocampal volume and context memory, $R^2$=.22, d.f.=15, p=.06, but no relationship between hippocampal volume and item memory, $R^2$=.13, d.f.=15, p=.16. Thus, the patient only correlations support the correlations performed across the entire participant population.

To assess whether the hippocampal volume loss in our patient group was selective to the hippocampus and did not affect extra-hippocampal MTL regions we analysed parahippocampal volume data (see Supplementary Experimental Procedures) from a subset of participants for which FreeSurfer volume estimates were available (11 Controls, 14 Patients). We first correlated hippocampal volume with parahippocampal volume, finding no significant correlation, $R^2$=.10, d.f.=23, p=.13. We also compared parahippocampal volumes across patients (mean: 9213mm$^3$; SD: 1836) and controls (mean: 9982mm$^3$; SD: 1754), finding no significant difference, $t(23)$ = .81, p=.43. Thus, the patients presented with selective hippocampal volume reductions.

Finally, we correlated item and context memory performance with parahippocampal and hippocampal volumes for this subset of participants. As in the main analyses, we found a correlation between hippocampal volume and context memory, $R^2$=.29, d.f.=23, p<.01, but not item memory, $R^2$=.09, d.f.=23, p=.15. The correlation with parahippocampal volume showed the reverse pattern, with a significant correlation with item memory, $R^2$=.18, d.f.=23, p<.05, but not context memory, $R^2$=.10, d.f.=23, p=.13. Thus, only the hippocampus correlated with context

memory performance. The item memory – parahippocampal volume correlation further strengthens the lack of correlation between hippocampal volume and item memory. In other words, our null result cannot be due to a ceiling effect for item memory as performance does correlate within an extra-hippocampal MTL region.

**Confidence Judgments**

Following the old/new item recognition judgment, participants were required to judge how sure they were regarding their memory judgment. Specifically, when the item was judged "new" they were required to respond "not sure" or "sure" as to the "new" status. If the item was judged "old" they were required to respond "not sure", "sure" or "very sure" about their ability to retrieve the associated scene stimulus. In other words, this judgment was related to the participants' subjective ability to retrieve the context relating to an old item.

Following a "new" judgment, the mean percentage of trials for "not sure" was 12% (SD 12%) and for "sure" was 88% (SD 13%) across participants. No relationship was seen between hippocampal volume and percentage "sure", $R^2$<.01, d.f.=29, p=.80, nor was a difference seen between patients and controls in percentage "sure" responses, $t(29) = .66$, p=.51. Table S2 presents the percentage CRs for "not sure" and "sure" responses across patients and controls (4 patients and 1 control removed due to no "not sure" responses). CR rates were higher for "sure" (mean 89%; SD 9%) than "not sure" (mean 59%; SD 34%) responses, $t(25) = 4.51$, p<.001. This increase in CR rate with increased confidence (i.e., CR "sure" – CR "not sure") was not related to hippocampal volume, $R^2$<.01, d.f.=24, p=.87, nor was a difference seen between patients and controls, $t(24) = .77$, p=.45.

Following an "old" judgment, the mean percentage of trials for "not sure" was 15% (SD 22%), for "sure" was 33% (SD 27%) and for "very sure" was 49% (SD 29%). No relationship was seen between hippocampal volume and percentage "not sure", $R^2$=.01, d.f.=29, p=.56, percentage "sure", $R^2$=.08, d.f.=29, p=.13, and percentage "very sure" $R^2$=.04, d.f.=29, p=.29. Furthermore, no difference was seen between patients and controls for percentage "not sure", $t(29) = .11$, p=.91, "sure", $t(29) = .37$, p=.72, and "very sure", $t(29) = .37$, p=.71, responses. Table S2 presents percentage item Hits for "not sure", "sure" and "very sure" responses across patients and controls. Hit rates were higher for "sure" (mean 83%; SD 20%) than "not sure" (mean 48%; SD 32%) responses, $t(30) = 5.37$, p<.001, and for "very sure" (mean 94%; SD 10%) than "sure" responses, $t(30) = 3.55$, p<.001. We could see no correlation between hippocampal volume and the increase in Hit rate between "not sure" and "sure" responses (i.e., Hit "sure" – Hit "not sure"), $R^2$=.03, d.f.=29, p=.32, nor was a difference seen between patients and controls, $t(29) = 1.08$, p=.29.

Thus far, we have shown that participants' confidence judgments were related to their item memory performance and that this relationship between subjective and objective memory status was unrelated to hippocampal volume. Next we assessed the relationship between context memory performance and confidence judgments. Table S2 presents percentage context Hits for "not sure", "sure" and "very sure" responses across patients and controls. Similar to item recognition performance, we saw a higher hit rate for "sure" (mean 36%; SD 27%) than "not sure" (mean 8%; SD 14%) responses, $t(30) = 6.70$, p<.001, and for "very sure" (mean 43%; SD 20%) than "sure" responses, $t(26) = 4.27$, p<.001. In contrast to the item recognition results, we saw a correlation between hippocampal volume and the increase in source hits between "not sure" and "sure" responses (i.e., Hit "sure" – Hit "not sure"), $R^2$=.15, d.f.=29, p<.05. However, the patient (mean 23%; SD 21%) vs. control (mean 33%; SD 24%)

comparison was not significant, $t(29) = 1.32$, $p=.20$. Despite this latter result, the correlational analysis revealed a relationship between hippocampal volume and accuracy of confidence judgments – the greater the hippocampal volume the greater the increase in context accuracy between "not sure" and "sure" confidence judgments.

## 3D SPM Analyses

### 300–350 Occipitotemporal and 350-400 Frontotemporal MEG Effect

The 3D SPM analysis revealed two distinct clusters between 300-400ms (as reported in the main analyses). Figure S1 presents the 3D SPM plots highlighting these two distinct effects.

As in the behavioural analyses, we formally compared the correlations between hippocampal volume and the frontotemporal and occipitotemporal effect by converting R-values to Z-values. This revealed a trend for a difference ($Z=1.27$, $p=.10$ one-tailed). A 2x2 (Frontotemporal vs. Occipitotemporal x Patients vs. Controls) ANOVA on the hits vs. CRs difference revealed a trend for an interaction, $F(1, 29) = 3.75$, $p=.06$. Thus, both analyses show trends in the expected direction, supporting the significant interaction seen in the behavioural results.

Our conclusion that the 500-600ms context-memory effect is reliant upon the early 350-400ms frontotemporal item-effect is supported by the lack of either effect in the Patient group (Figure 4). Nonetheless, we performed a further analysis whereby we median split individual trial ERF data based on the size of the 300-350ms occipitotemporal effect and the 350-400ms frontotemporal effect (relative to each participants average CR effect) and subsequently analysed the size of the later 500-600ms frontotemporal effect based on these splits. We reasoned that if the 350-400ms frontotemporal effect was necessary for future context retrieval we should see a larger 500-600ms hits vs. CRs effect for trials that showed a large 350-400 frontotemporal effect. We did indeed see this pattern when splitting based on the 350-400ms frontotemporal effect, $t(13) = 6.00$, $p<.001$, but not the 300-350ms occipitotemporal effect, $t(13) = 1.61$, $p=.13$, further supporting our main conclusion.

### 500–600 ms Frontotemporal MEG Effect

The 500-600ms time-window that showed a context effect in the Controls but not Patients was selected *a priori* based on previous EEG results showing significant context modulation within this time period. We also searched for differences between context hits and misses over the two sensors of interest (frontotemporal and occipitotemporal) across the entire epoch. Performing t-tests at each time point, we searched for contexts hits vs. misses differences that were significant for more than 10 time-points in a row (alpha = 0.05, probability of 10 significant sequential timepoints in 361 samples <.001). The only time-period to show a significant effect across the frontotemporal sensors in the Controls was between 480-520ms, a period overlapping our 500-600ms timewindow. No further significant time-periods were seen in either the Patients or Controls in the frontotemporal or occipitotemporal sensors.

### 700–750 ms Frontotemporal MEG Effect

The 3D SPM analysis revealed a third significant cluster from 720ms to 760ms over the same frontotemporal sensors as the earlier frontotemporal effect reported in the main analyses (see Figure S2A). Here, a difference between Hits and CRs was only present in the Controls, $t(13) = 3.20$, $p<.01$, but not the Patients, $t(16) = 1.07$, $p=.30$. Given the sustained nature of the frontotemporal effect, this later cluster is likely to

reflect the same underlying effect as the earlier frontotemporal effect (with a present, but subthreshold effect between the two temporally distinct clusters). The sustained nature of this effect is highlighted in Figure S2C, starting at approximately 300ms and continuing throughout the rest of the epoch. However, the 700-750ms timewindow Hits vs. CRs effect over the same sensors was not correlated with hippocampal volume, $R^2$=.09, d.f.=29, p=.11.

**Further 3D SPM Analyses**

Given we did not want to bias any of the correlations between MEG effects and hippocampal volume, we focussed the main SPM analyses on the main effect of hits vs. CRs, collapsed across Controls and Patients. Thus, we did not search for any effect using the 3D SPM approach that showed a significant interaction between the main effects of hits vs. CRs and Controls vs. Patients. This interaction term revealed several distinct clusters from 450-1000ms over the same right frontotemporal sensors, with the largest cluster peaking at 850ms (p<.001 uncorrected; see Figure S3A).

The topographies of this effect over the peak right frontotemporal sensors (N=4) split by patients and controls show a negative effect (i.e., CRs>Hits) in the controls but a positive effect in the patients (Figure S3B). Both the negative effect in the controls and the positive effect in the patients reached significance in an 820-870ms time-window, based on the 850ms peak shown in the SPM analysis (Figure S3D). Finally, the difference between Hits and CRs within this time-window correlated with hippocampal volume, $R^2$=.20, d.f.=29, p<.05.

**Table S1, Related to Figure 1.**

| | Item Memory | | | | | Context |
|---|---|---|---|---|---|---|
| | Hits | Misses | CRs | FAs | Pr | Hits |
| Patients | 83.3 (9.1) | 14.8 (7.8) | 82.4 (12.4) | 14.4 (10.5) | 68.8 (15.8) | 33.1 (13.5) |
| Controls | 84.4 (13.6) | 13.5 (12.1) | 87.6 (10.6) | 8.2 (6.3) | 76.2 (16.5) | 54.9 (20.5) |

Mean percentage (plus standard deviations) for hits, misses, correct rejections (CRs) and false alarms (FAs) as well as the Pr (hits – false alarms) measure for the word old/new judgment (item memory) and the conditional probability of Hits for the context judgment (Context) across the Patient and Control groups.

**Table S2, Related to Figure 1**.

| | | Patients | Controls |
|---|---|---|---|
| **Item Memory** | | | |
| *"new" responses* | | | |
| CRs | not sure | 52.5 (40.0) | 67.5 (25.1) |
| | sure | 87.6 (7.7) | 90.3 (11.0) |
| *"old" responses* | | | |
| Hits | not sure | 44.9 (31.5) | 55.1 (33.8) |
| | sure | 85.2 (11.0) | 81.7 (27.9) |
| | very sure | 92.6 (11.9) | 96.0 (5.0) |
| **Context Memory** | | | |
| Hits | not sure | 6.1 (11.6) | 15.3 (21.1) |
| | sure | 28.7 (22.3) | 48.5 (32.3) |
| | very sure | 34.5 (13.6) | 57.6 (25.8) |

Mean percentage (plus standard deviations) for item memory correct rejections (CRs) when an item was judged "new" across "not sure" and "sure" confidence judgments, item memory Hits when an item was judged "old" across "not sure", "sure" and "very sure" confidence judgments, and context memory hits when an item was judged "old" across "not sure", "sure" and "very sure" confidence judgments in the Patient and Control group.

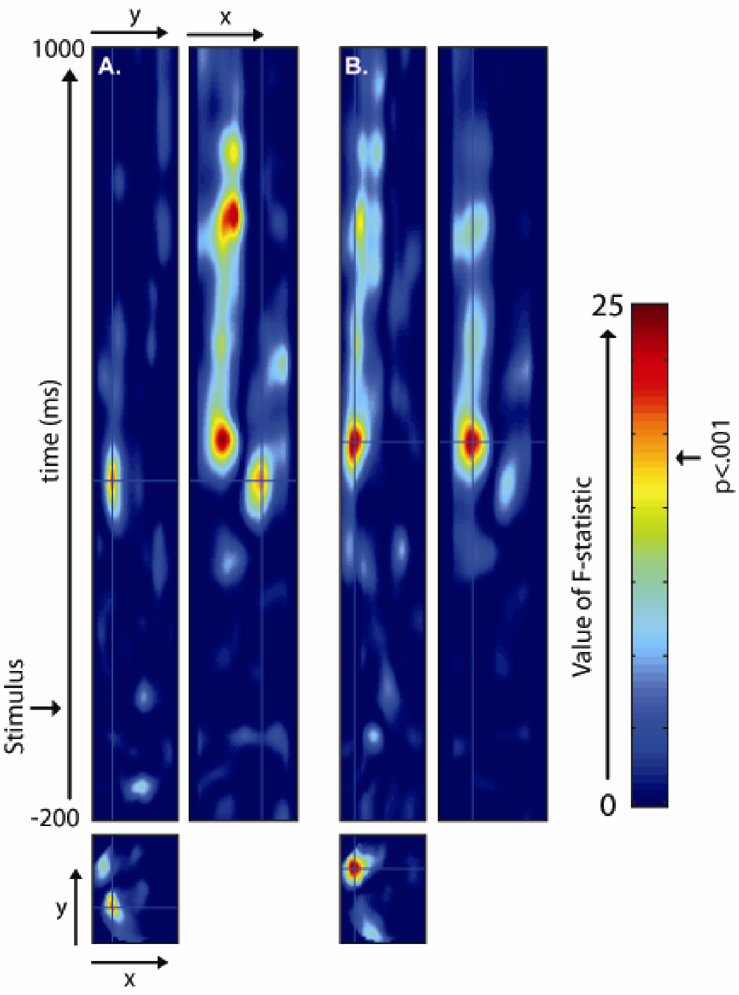


**Figure S1, Related to Figure 3. Magnetoencephalographic Effects across All Sensors and Time Points**

Unthresholded space-time Statistical Parametric Maps (SPMs) for the F-test Hits vs. CRs (collapsed across Controls and Patients) with (A) the crosshair located on the left occipitotemporal maximum at 330ms and (B) the crosshair on the left frontotemporal maximum at 390ms. The three images in each panel represent the orthogonal planes (y-t, x-t, x-y) through the 3D image at the location of the crosshair. Colour-maps show F-values with approximate threshold for p<.001 uncorrected.

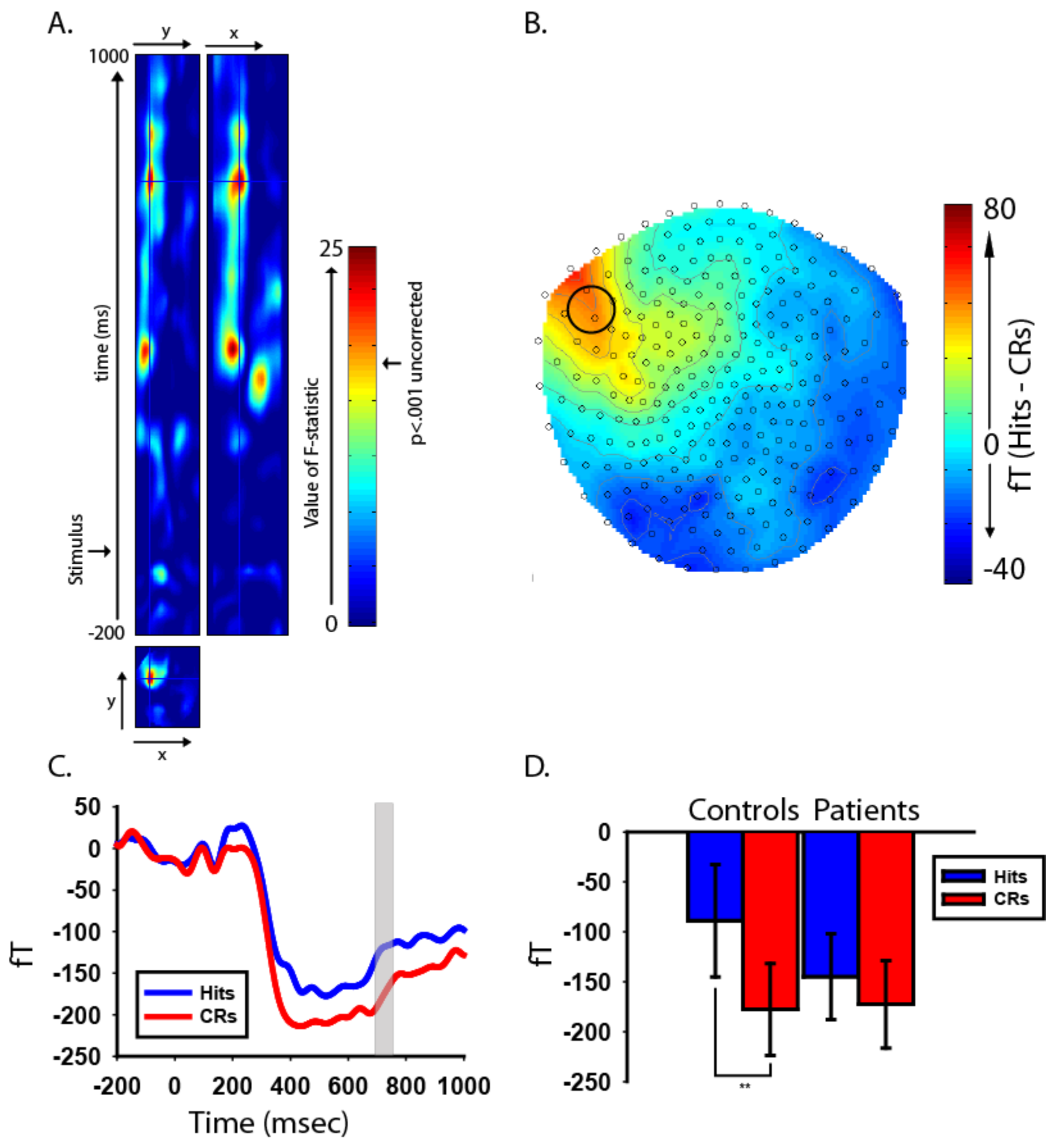


**Figure S2, Related to Figure 3.**
(A) Unthresholded space-time SPM for F-test Hits vs. CRs (collapsed across Controls and Patients) with crosshair located on the left frontotemporal maximum at 740ms. The three images in each panel represent the orthogonal planes (y-t, x-t, x-y) through the 3D image at the location of the crosshair. Colour-maps show F-values with approximate threshold for p<.001 uncorrected.
(B) Topography of the difference in fT between Hits and CRs at the mid-timepoint in the 700-750ms timewindow with the black circle highlighting the sensors selected for further analyses.
(C) ERFs for Hits and CRs averaged across the peak sensors with the 700-750ms timewindow highlighted in grey (collapsed across Patients and Controls).
(D) Timewindow analyses for the average (fT) within the 700-750ms timewindow, plotted separately for Controls and Patients. Error bars show +/- 1 standard error of the mean for each condition; **p<.01.

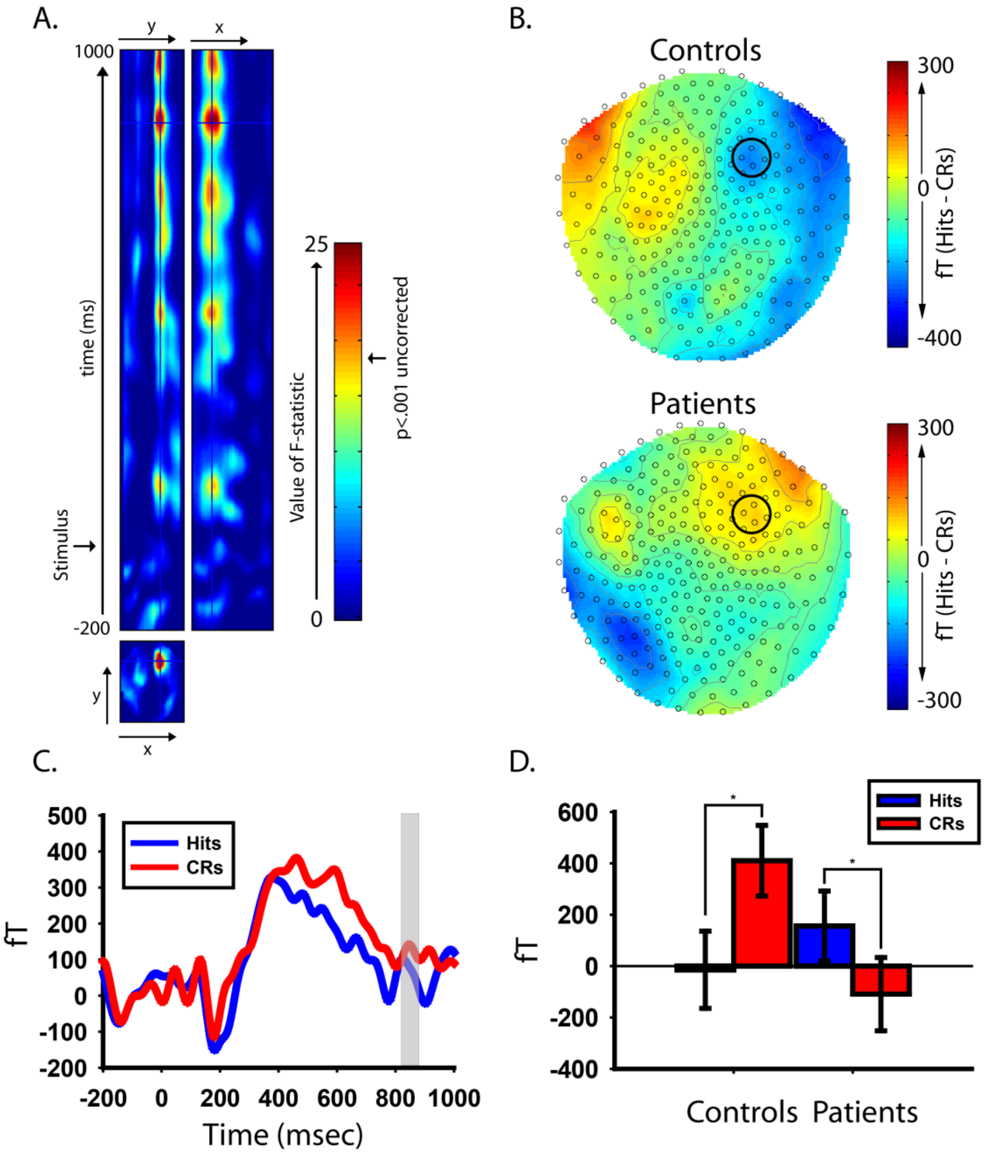


**Figure S3, Related to Figure 3.**
(A) Unthresholded space-time SPM for F-test Hits vs. CRs x Patients vs. Controls with crosshair located on the right frontotemporal maximum at 850ms. The three images in each panel represent the orthogonal planes (y-t, x-t, x-y) through the 3D image at the location of the crosshair. Colour-maps show F-values with approximate threshold for $p<.001$ uncorrected.
(B) Topography of the difference in fT between Hits and CRs at the mid-timepoint in the 820-870ms timewindow for patients and controls with the black circle highlighting the sensors selected for further analyses.
(C) ERFs for Hits and CRs averaged across the peak sensors with the 820-870ms timewindow highlighted in grey (collapsed across Patients and Controls).
(D) Timewindow analyses for the average (fT) within the 820-870ms timewindow, plotted separately for Controls and Patients. Error bars show +/- 1 standard error of the mean for each condition; $*p<.05$.

## Supplemental Experimental Procedures

### Participants

Seventeen patients (8 male, mean age 18.2 years, range 10-35 years) participated. This patient group included 7 participants with mixed etiologies resulting in a diagnosis of developmental amnesia (DA). Of the remaining 10 participants, 6 were born at term with acute hypoxemic respiratory failure (AHRF), requiring extracorporeal membrane oxygenation (ECMO), and 4 were born at term with transposition of the great arteries (TGA) and surgically treated during the neonatal period with the arterial switch operation (ASO).

Fourteen healthy controls (6 male, mean age 18.6 years, range 11-35 years) also participated. There was no significant difference between the patient and control groups in terms of age, $t(29) = .14$, $p=.86$. Controls were recruited either from London schools through invitation letters sent to the families via the head teacher, or through friends of the patients with DA. All control participants were attending mainstream schools, and were not receiving any special educational help. The adult Controls were in employment.

All young participants and their parents, as well as the adult Controls gave informed consent to participate in accordance with the regulations of the local research ethics committee (ethics code: 05/Q0502/88). As shown in Figure 1C, the Patient and Control groups were comparable in terms of IQ (full-scale IQ using Wechsler's Intelligence Scale for Children (WISC-IV) or Wechsler's Adult Intelligence Scale (WAIS-III), literacy (mean across Word Reading, Reading Comprehension and Spelling subtests of Wechsler's Individual Achievement Test (WIAT) and numeracy (mean across Numerical Operations and Mathematical Reasoning subtests of WIAT). In line with their known episodic memory deficits, the Patient group's memory quotient (MQ – General Memory composite score from Children's Memory Scale (CMS) or Wechsler's Memory Scale (WMS-III)) was impaired relative to Controls.

### Procedure

The experiment consisted of 6 Study-Test cycles (see Figure 1A). At Study, participants were presented with 10 word-scene pairs and were asked to respond to whether the word denoted a living or non-living object. The trial sequence started with a fixation cross for 500ms followed by a scene for 3000ms. A fixation cross was then presented for 1500ms followed by presentation of the same scene with its paired word presented in red for 3000ms. Following word-scene presentation participants were given 2000ms to make their living/non-living judgment. Trials ended with a blank screen presented for 1600, 1800, 2000 or 2200ms, randomly selected. Following the Study phase, participants were required to perform a brief block of arithmetic to minimize the possibility of them actively rehearsing the word-scene pairs prior to the Test block (8 problems, 15secs per problem).

At Test, participants were presented with 10 Old and 10 New words and required to judge the old/new status of each word. If a New classification was given, they judged whether they were "not sure" or "sure" about this new judgment. If an Old classification was given, they judged whether they were "not sure", "sure" or "very sure" they could recollect the scene that was paired with the word at Study. Following this judgment, the correct scene was presented alongside two incorrect scenes and a blank square in the four corners of the screen. Participants were instructed to choose the scene originally paired with the word (or select the blank

square if they thought it wasn't present). The two incorrect scenes had also been presented at Study (paired with different words) therefore participants had to base their selection on the word-scene pair. Trials started with a 500ms fixation followed by an Old or New word for 2500ms and another fixation cross for 500ms. Participants were then given 2000ms to make their old/new judgment followed by 2000ms for their confidence judgment. If the word was categorised as Old, they were then given 4000ms for scene selection. Trials ended with a blank screen presented for 1600, 1800, 2000 or 2200ms, randomly selected.

**Medial Temporal Lobe Volume Data**

Figure 1B presents individual hippocampal volumes across all participants in rank order. As can be seen, a linear relationship is seen across the participants, with little evidence for a step-change in hippocampal volume between patients and controls. To assess this relationship more formally, we fitted a linear and sigmoid function to the ranked data, reasoning that if a step-change were present between groups a sigmoid function should be a better fit to the data than a linear function. Whereas a linear function explained a large proportion of the variance ($R^2$=.95), a 3-parameter sigmoid function explained only a small proportion of the variance ($R^2$=.48). Thus, we could find no evidence for a step-change in hippocampal volume between patients and controls. Volumes for bilateral extra-hippocampal regions were also estimated using automated FreeSurfer analyses. We used the Parahippocampal FreeSurfer region from these analyses as an estimate of extra-hippocampal medial temporal lobe volume given this region encompasses much of the parahippocampal gyrus as well as more anterior regions including the perirhinal cortex.

**MEG Data Preprocessing**

The continuous MEG data from each Test session were high-passed filtered at 0.1Hz using a 5$^{th}$-order Butterworth filter and downsampled to 300Hz. The data were subsequently epoched from -200ms to 1000ms (with 800ms padding at the beginning and end of each epoch), low-pass filtered at 16Hz, and baseline corrected from -200ms to 0ms. Participant-specific averages were calculated across trials within each condition.

**3D SPM Analysis**

The 2D channel space was created by a spherical projection of the 275 MEG sensors onto a plane, followed by a linear interpolation to a 32x32 pixel grid. The time dimension consisted of 361 3.3ms samples in each epoch. Two 3D space x times images per participant (item recognition hits vs. CRs) were entered into a GLM with four experimental conditions (relating to Hits vs. CRs across the Control and Patient groups) and 31 additional subject effects.

**Correlative Analysis Approach**

The primary aim of this study was to assess the extent to which the hippocampus contributes to item and context memory. Furthermore, we aimed to associate hippocampal volume with mnemonic event-related MEG components, providing further evidence for a dissociation between item and context memory. With this in mind, the main analyses correlated our behavioural and MEG measures with hippocampal volume across the entire participant sample (i.e., patients and controls). We reasoned such a correlative approach may potentially be more sensitive than traditional patient vs. control comparisons given the linear distribution of

hippocampal volumes across the participant sample. Nonetheless, we report more traditional patient vs. control comparisons alongside the correlational analyses to increase the clarity of results.
